# Agricultural Water Use Efficiency and Rebound Effect: A Study for China

**DOI:** 10.3390/ijerph18137151

**Published:** 2021-07-04

**Authors:** Hang Xu, Rui Yang, Jianfeng Song

**Affiliations:** 1 College of Economics and Management, Northwest A&F University, Yangling 712100, China; xuhang@nwafu.edu.cn; 2 College of Humanities and Development Studies, China Agricultural University, Beijing 100083, China; rui@cau.edu.cn

**Keywords:** agricultural water use efficiency, agricultural water use, rebound effect, China

## Abstract

Agricultural water use accounts for the largest proportion of water withdrawal, so improving agricultural water use efficiency is an important way to alleviate water shortage. However, the expected water saving by the improved agricultural water use efficiency may be offset by the rebound effect, which means the goal of water saving by improving agricultural water use efficiency is not achieved. Based on the definition of the rebound effect of agricultural water use, this paper first uses a fixed model to measure the causal effect of agricultural water use efficiency on agricultural water use to analyze the agricultural water rebound effect, then analyses the heterogeneity and mechanism of the effect of agricultural water use efficiency on agricultural water use with the panel data from 30 provinces or cities in China from 2000 to 2017. The results show that, firstly, the agricultural water use efficiency has a significant negative effect on agricultural water use, but the average agricultural water rebound effect is 88.81%. Secondly, the effect of agricultural water use efficiency on agricultural water use is heterogeneous, in which the improvement of agricultural water use efficiency in humid or major grain-producing areas will have a lower agricultural water rebound effect. Finally, agricultural water use efficiency can affect agricultural water use through planting area and planting structure. An increase in agricultural water use efficiency will expand the planting area to increase water use. However, this will change the planting structure to decrease water use. The implication for agricultural water management is that the irrigation agricultural scale has to be controlled under the condition of available water resource, while improving agricultural water use efficiency.

## 1. Introduction

In the context of finite supply and climate change, the growing demand for freshwater is one of the most significant challenges facing humanity [1]. Agriculture is the largest user of water globally. Thus, promoting agricultural water use efficiency is a basic and fundamental approach to alleviating water scarcity because of regional differences in water resources endowment. Theoretical studies generally agree that high-efficiency water-saving technology can ideally save a lot of water resources [2]. However, over the past few decades, water conservation practices in many places did not result in reduced agricultural water use as expected, though agricultural water use efficiency had increased significantly [3]. This phenomenon is also happening in China, which is a country with water scarcity and one of the 13 water-poor countries in the world. The total freshwater resources of China account for 6% of the world’s water resources, but the per capita amount is only one-quarter of the world’s average level. In 1998, the Chinese government began to formulate policies to promote water saving and vigorously develop water-saving agriculture, thus greatly improving agricultural water use efficiency. However, China still faces a huge water shortage. Therefore, the expected goal of reducing the total amount of water used in agriculture by increasing agricultural water use efficiency has not been achieved, which is usually referred to as the “rebound effect” [4].

Agricultural water use efficiency programs, widely promoted by many governments, may result in undesired outcomes such as rebound effects [5]. The rebound effect, also known as the Jevons paradox [6], was first identified by Jevons in 1866 in a seminal study of coal use during the Industrial Revolution [1], revealing that increases in the energy efficiency did not decrease but instead increased coal use. The rebound effect also occurs in water use [7]. Similar to energy rebound, improvements in water-saving irrigation technology can improve the efficiency of agricultural water use, but the expected water-saving effect may be offset by additional water use. Most studies have focused on the adoption of water-saving irrigation technology, but few works of literature have further analyzed the usually overlooked effects of water-saving technology and efforts. Ward and Pulido-Velazquez [8] found that irrigation water use decreased after the improvement of water-saving technology when a field scale is considered; however, they found that irrigation water use increased after the improvement of water-saving technology when they further analyzed the watershed scale. Peterson and Ding [9] believe that the reason for the decrease in irrigation water use with the improvement of water-saving technology is the decrease in irrigation area, so the increase in irrigation efficiency may increase or decrease irrigation water use. Fishman et al. [10] also found that the potential savings of underground water under the improvement of water-saving technology was offset by the additional water use because of the expansion of irrigated area. Grafton et al. [3] pointed out the paradox that the improvement of irrigation efficiency failed to reduce agricultural water use under the current subsidy policy for water-saving technology. Therefore, policy makers need to reconsider the current agricultural water-saving policy.

Although some studies have focused on the water-saving irrigation technology adoption [11,12,13], few have analyzed the effect of the improvement of water-saving irrigation technology on water use/withdrawal from the perspective of water rebound effect. Pfeiffer and Lin [14] found that irrigation water use increased after the implementation of more efficient irrigation technology, clearly pointing out that this was a rebound effect greater than 100%. This was the first time that the concept of “rebound effect” was used to express the water-saving effect. While water rebound is similar to energy rebound, the definition varies. One view emphasizes that it can be defined as rebound effect when the water-saving is partially offset by new water use [6,14,15]. The other view believes that the phenomenon that the improvement of irrigation technology increases rather than reduces total water use is what is called the rebound effect [5,16]. Although there are some differences between the two views, they both agree that the rebound effect of irrigation water is a measure of the degree to which the water-saving is offset, while the second view only emphasizes the situation that the expected water-saving amount is completely offset, which can be regarded as a special case of the first view. Some researchers have quantitatively measured the rebound effect [4,5,6,7,14,15,16,17]. However, measurements of the rebound effect do not reflect the casual effect of agricultural water use efficiency on water use because they ignore other factors, such as climatic and economic factors, which will lead to misunderstanding of the agricultural water rebound effect. Ajaz et al. [18] highlighted the climatic and economic aspects as factors governing the expansion of irrigated area due to the increase in water use efficiency and water conservation practices.

Although the literature on the water rebound effect is increasing, details on the mechanism of water rebound effect remain scarce. According to the existing literature, the mechanism of the rebound effect in agricultural water use can be summarized according to two aspects. The first is called the hydrological mechanism, which is always discussed in the hydrological literature. The hydrological mechanism means that agricultural water use efficiency improvement with advanced technology can match the crop’s need of water more precisely, making it easy to achieve full irrigation and, thus, agricultural water use may increase along with crop yield [8,19,20,21,22,23,24,25,26]. The second is economic mechanism, in which the agricultural water use efficiency improvement makes water resource cheaper for producing the same amount of crops, and farmers would change their planting behaviors in response to the improvement in agricultural water use efficiency [1,5,7,14,27,28,29,30,31]. Although the two aspects have been discussed in the literature, the mechanism of agricultural water rebound effect is still ambiguous, and empirical studies on this are required.

The improvements in agricultural water efficiency were considered a key way to ease the water scarcity, but the studies on agricultural water rebound effect make it controversial to subsidize agricultural irrigation technology to improve the water use efficiency. In order to reasonably manage water resources in the agricultural sector, most of the existing literature focuses on measuring the magnitude of agricultural rebound effect, but the existing literature is not based on causality and ignores the mechanism of the rebound effect. Therefore, this paper has two main objectives. The first is to estimate the average agricultural water rebound effect in China by analyzing the causal effect of agricultural water use efficiency on agricultural water use. The second is to conduct an empirical analysis of the mechanism of the agricultural water rebound effect, mainly based on the economic mechanism through planting area and planting structure, which will be more beneficial to establishing agricultural water-saving policies.

This paper applies a fixed effect model to estimate the causal effect of agricultural water use efficiency on agricultural water use to understand agricultural water rebound effect using 2000–2017 panel data of 30 provinces or cities in China (not including Hong Kong, Macao, Taiwan and Tibet). The present work is organized as follows: Section 2 defines the rebound effect of agricultural water use and constructs research hypotheses through theoretical analysis. In Section 3, details of the materials and methods are provided. The results of agricultural water rebound effect are introduced in Section 4. The magnitude and mechanism of the agricultural water rebound effect are discussed in Section 5. Finally, a summary is provided and some conclusions are highlighted in Section 6.

## 2. Theoretical Analysis of Agricultural Water Rebound Effect

### 2.1. Definition

Since the theory of the energy rebound effect was first proposed, two different methods of estimating the rebound effect have been developed. One method is to directly compare the demand before and after the improvement of efficiency, and the other method focuses on elasticity as a proxy variable [32,33,34,35,36,37,38]. However, only the method of elasticity can be used to estimate the causal effect of agricultural water use efficiency on water use.

According to Fei et al. [4] and Song et al. [7], the agricultural water rebound effect (WRE) can be defined as:

(1) $$WRE=1+W_{\eta },$$

where WRE denotes agricultural water rebound effect, and $W_{\eta }$ denotes the efficiency elasticity of agricultural water use. The ideal state is that a 1% increase in agricultural water use efficiency would lead to 1% reduce in water use. However, some part of the expected water saving may be offset due to the existence of rebound effect. A WRE of 10% indicates that 10% of the expected water savings are offset by increased water use. Additionally, if the WRE is over 100%, the agricultural water use efficiency improvements can even increase water use, which is called the “backfire effect”.

To obtain WRE, $W_{\eta }$ should be calculated. $W_{\eta }$ also denotes the offset proportion, which can be defined as:

(2) $$W_{\eta }=\frac{\partial lnW}{\partial ln\eta },$$

where lnW denotes the natural logarithm of the agricultural water use, and lnη denotes the natural logarithm of the agricultural water use efficiency. $W_{\eta }$ also denotes the causal effect of agricultural water use efficiency on agricultural water use.

To obtain WRE, the agricultural water use efficiency can be calculated by meta-frontier data envelopment analysis method, then we can estimate the causal effect of agricultural water use efficiency on agricultural water use by fixed effect model to get the efficiency elasticity of agricultural water use.

### 2.2. Research Hypothesis

Increased agricultural water use efficiency often means that water consumption by crops is increased because the service more precisely and uniformly matches the water needs of a crop [39]. Thus, water use at the field level will decrease as expected if the water consumption is constant. However, water consumption at the field level may increase. Many researchers have observed this phenomenon of increasing water consumption, and some of them regard it as a rebound effect of irrigation water [6,40]. However, this phenomenon may just be a technical property of irrigation efficiency improvements, which is called hydrological mechanism of rebound effect. The hydrological mechanism indicates that improved agricultural water use efficiency increases water consumption by crops because the improved irrigation system more precisely and uniformly matches the water needs of a crop [8,19,20,22,23,24,25,26]. The technical property is the basis for the economic mechanism of rebound effect because the precise irrigation makes it cheaper to use water in production.

When agricultural water use becomes relatively cheaper, its use at the field level may not decrease as expected after agricultural water use efficiency improvements [19,41,42,43]. The water use may increase due to farms’ behavioral adjustments in response to improved agricultural water use efficiency at the field level. The farms will first adjust the planting area and planting structure, then irrigating the crops for profits. Hence, the key factors of the behavior adjustment of farmers are the change of planting area and planting structure in response to agricultural water use efficiency improvements, resulting in more water use, which can be seen as the economic mechanism of the rebound effect. To sum up, the hydrological mechanism represents the technical property of irrigation efficiency improvements, which makes it relatively cheaper for water use. Moreover, the main additional agricultural water use is due to the change in the sown structure and expansion of sown area in response to the improved agricultural water use efficiency, which will lead to more water demand. The research hypothesis is as follows:

**Hypothesis** **1** **(H1).** *The agricultural water use efficiency will reduce agricultural water use, but it cannot be reduced in equal proportion.*

**Hypothesis** **2** **(H2).** *The agricultural water use efficiency will affect agricultural water use through planting area and planting structure.*

## 3. Materials and Methods

### 3.1. Method for Agricultural Water Use Efficiency

**Figure 1:**
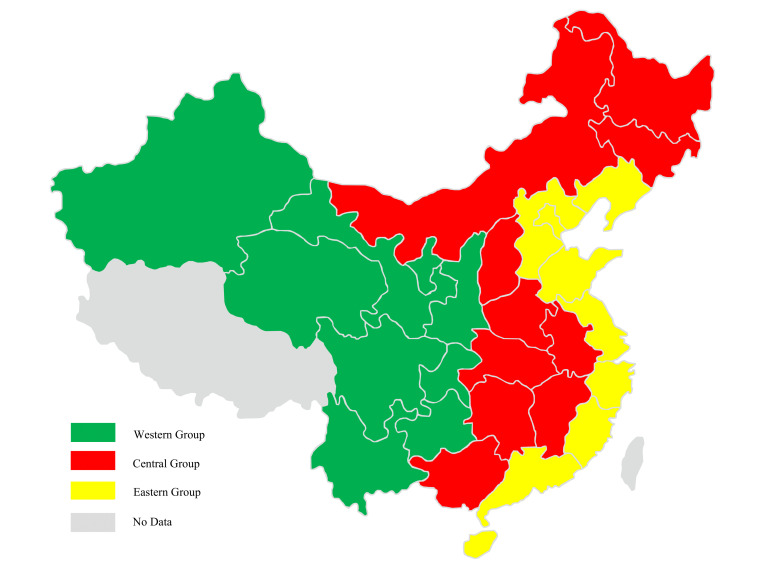
The groups based on technical heterogeneity of selected provinces in China.

The meta-frontier data envelopment analysis (DEA) method, based on technical heterogeneity, was used to estimate the agricultural water use efficiency, which is consistent with the actual situation of China’s economy [4]. Considering the geographical location, resource endowment and economic development level of each province in China, the selected 30 provinces are divided into three regions, namely the eastern group, the central group, and the western group. This grouping method has been widely used in studies of problems in China [44,45,46]. The grouping results are shown in Figure 1 and the details can be seen in Table 1.

Assuming that a production system can be classified into H groups according to certain standards, and each group contains J decision-making units (j = 1, 2, …, J), applying M kinds of inputs to produce one kind of output. In this paper, capital *K*, labor *L*, and water resources *W* are the main inputs, and agricultural GDP is the output *Y*. Then, the production technology T of the group *h* can be defined as follows:

(3) $$T^{h}=\left\{\left(K,L,W,Y|\delta_{h}\right):(K,\;L,\;W)\;\mathrm{can}\;\mathrm{produce}\;Y\;\mathrm{with}\;\mathrm{technology}\;\delta_{h}\right\}$$

The groups are assumed to use different technologies each year, then different production technology frontiers are required. Based on the Equation (3), the distance function of group *h* in year *t* is defined as follows:

(4) $$D^{ht}\left(K,L,W,Y\right)=sup\left\{\rho :\left(K,L,W/\rho ,Y\right)\in T^{ht}\right\}$$

The economic implication of the distance function is to calculate the maximum degree ρ of potential water saving under given technology, holding other input factors (capital *K* and labor *L*) and output *Y* unchanged. The agricultural water use efficiency based on the given technology frontier can be written as:

(5) $$MTFEE^{ht}=1/D^{ht}\left(K,L,W,Y\right)$$

For simplicity, according to Fei et al. [4], the agricultural water use efficiency of each decision-making unit (DMU) under technology of the group can be calculated as:

(6) $$GGLPI=\frac{\mathrm{Target}\;\mathrm{water}\;\mathrm{use}\;\mathrm{based}\;\mathrm{on}\;\mathrm{the}\;\mathrm{front}\;\mathrm{frontier}}{\mathrm{Actual}\;\mathrm{water}\;\mathrm{use}}$$

As the common frontier is the data envelope of group frontiers, the group frontiers (h = 1, 2, ⋯, H) are enveloped to construct a common frontier. Then, the agricultural water use efficiency index (MGLPI) based on the common frontier is symbolized as [4]:

(7) $$MGLPI=\frac{\mathrm{Target}\;\mathrm{water}\;\mathrm{use}\;\mathrm{based}\;\mathrm{on}\;\mathrm{the}\;\mathrm{common}\;\mathrm{frontier}}{\mathrm{Actual}\;\mathrm{water}\;\mathrm{use}}$$

Based on the above analysis, the agricultural water use efficiency (WUE) can be acquired by calculating the index (MGLPI), which can be defined as the ratio of the optimal input quantity of water to the actual one.

### 3.2. Estimating Method

Although agricultural water use efficiency is promoted by the national policy of the Chinese government, the programs are still related to the typical features of regions and other variables, which will also affect the agricultural water use. In order to avoid estimation errors caused by the influence of typical characteristics of different regions, the fixed effect model is adopted to study the causal effect of agricultural water use efficiency on agricultural water use. In addition, some control variables are also considered. In order to test the mechanism of agricultural water rebound effect, mediator variables are used in the econometrics model [47,48]. Hence, the mediation model is considered, which can be defined as follows:

(8) $$lnWU_{it}=\alpha_{i}^{1}+\beta^{1}lnWUE_{it}+\gamma^{1}lnX_{it}+\epsilon_{it}^{1}$$

(9) $$lnME_{it}=\alpha_{i}^{2}+\beta^{2}lnWUE_{it}+\gamma^{2}lnX_{it}+\epsilon_{it}^{2}$$

(10) $$lnWU_{it}=\alpha_{i}^{3}+\beta^{3}lnWUE_{it}+\gamma^{3}lnX_{it}+\mu lnME_{it}+\epsilon_{it}^{3}$$

where *i* denotes province and *t* denotes year. $lnWU_{it}$ is the natural logarithm of the agricultural water use. $lnWUE_{it}$ is the natural logarithm of the agricultural water use efficiency, which belongs to the core explanatory variable. In order to avoid the endogeneity of agricultural water use efficiency, apart from considering regional fixed effect, we also choose a series of variables which may be associated with agricultural water use and agricultural water use efficiency. The control variables are the effective irrigation area, rainfall, and drought area, which are all logarithmically processed and denoted by $lnX_{it}$. $lnME_{it}$ denotes the natural logarithm of the mediator variables, which includes planting area and planting structure. $\alpha_{i}^{1}$, $\alpha_{i}^{2}$, and $\alpha_{i}^{2}$ are the province fixed effects. $\beta^{1}$, $\beta^{2}$, and $\beta^{3}$ are the corresponding coefficients of the $lnWUE_{it}$. $\gamma^{1}$, $\gamma^{2}$, and $\gamma^{3}$ are the corresponding coefficient vectors of $lnX_{it}$. μ is the corresponding coefficient of $lnME_{it}$. $\epsilon_{it}^{2}$, $\epsilon_{it}^{2}$ and $\epsilon_{it}^{3}$ are the residual terms.

The construction of the mediation model above has two purposes. Firstly, Equation (8) is used to estimate the efficiency elasticity of agricultural water use to get the average rebound effect of agricultural water in China. Secondly, Equations (9) and (10) are used to test the mechanism of the mediator variables in the effect of agricultural water use efficiency on agricultural water use after the estimation of Equation (8).

### 3.3. Variable Selection and Data Sources

In order to avoid errors in estimating the causal effect of agricultural water use efficiency on agricultural water use because of the possible omission of variables, the control variables which are related to the agricultural water use efficiency and agricultural water use are considered, including drought area, effective irrigation area, and rainfall. The agricultural water-saving policy implemented by the Chinese government is an important factor for the improvement of agricultural water use efficiency. The promotion of this policy will depend on regional drought conditions, so the agricultural water use efficiency will be related to rainfall and drought area. In addition, the promotion of agricultural water-saving policies also depends on water conservancy infrastructure, so the agricultural water use efficiency is also related to the effective irrigation area. Moreover, the drought area, effective irrigation area, and rainfall will also affect the agricultural water use. Based on this, the independent variable is agricultural water use efficiency, and the depend variable is agricultural water use, while the control variables are rainfall, drought area and effective irrigated area.

Agricultural water use efficiency (WUE) reflects the ratio of the optimal input quantity of water to the actual one in production. Agricultural water use (WU) mainly refers to the water resources used for agriculture. Apart from agricultural water use efficiency and agricultural water use data, three control variables are also included, which are rainfall, drought area, and effective irrigation area. Rainfall (RF) is the amount of rain that falls from the sky to the ground and accumulates on the surface of the water without evaporation, infiltration, or loss, which reflects regional drought and is also one of the important bases for the promotion of Chinese agricultural water-saving policies. Drought area (DA) refers to the area sown to crops that suffer from severe drought resulting in reduced production, which reflects the extreme drought situation in the region. Effective irrigated area (EIA) refers to the area of arable land with a certain water source, relatively flat land, and complete irrigation projects or equipment which can be normally irrigated in ordinary years. It is an important index reflecting the construction of farmland water conservancy in China and also the basis for the promotion of water-saving irrigation.

In the analysis, the mechanism of agricultural water rebound effect will be determined by testing the mediating effect in causality of agricultural water use efficiency affecting agricultural water use. According to the existing analysis on rebound effect, the planting area and planting structure will be mainly considered [5,14,30,31]. Planting area (PA) refers to the planted or transplanted area of the harvested crops on all the land (cultivated or non-cultivated) in the calendar year. The planting structure (PS) is mainly represented by the planting proportion of grain crops in the sown area of crops. Planting area (PA) and planting structure (PS) represent the behavior adjustment of farms in production.

In this paper, the panel data of the 30 provinces in China (not including Hong Kong, Macao, Taiwan and Tibet) from 2000 to 2017 were used as the research sample. The data of agricultural water use efficiency (WUE), which is calculated by meta-frontier data envelopment analysis (DEA) method, is from Fei et al. [4]. The data of agricultural water use (WU) and rainfall (RF) are from China Water Resources Bulletin. The data of drought area (DA) comes from China Rural Statistical Yearbook. The data of effective irrigated area (EIA) is mainly derived from China Water Yearbook. The data of planting area (PA) and planting structure (PS) were obtained from the statistical yearbooks of each province. The descriptive statistics of the variables are shown in Table 2.

## 4. Results

### 4.1. Rebound Effect

Equation (8) is estimated to get the efficiency elasticity of agricultural water use in order to calculate the average rebound effect of agricultural water in China. In the estimation model, the control variables were firstly excluded, then different control variables were separately added, and finally all control variables were added, specifically forming five models. The estimated results are shown in Table 3. It should be noted that the estimation model mainly adopts the fixed effect model, and each variable is logarithmically processed.

Table 3 can reflect the casual effect of agricultural water use efficiency on agricultural water use and the agricultural water rebound effect. The regional fixed effect was included in Model 1 without the control variables, and the regression results show that agricultural water use efficiency has a negative impact on agricultural water use at a significance level of 1%. However, agricultural water use would be reduced only by 0.1452% for each 1% increase in agricultural water use efficiency, which means the agricultural water rebound effect is 85.48%. In order to avoid possible estimation bias, control variables were considered in the remaining models. In Model 2, after the effective irrigation area was also added as the control variable, the influence of agricultural water use efficiency on agricultural water use was still significant at the level of 1%, but the absolute value of the estimated coefficient decreased relative to Model 1. In Model 3, after the drought area was also added as the control variable, the agricultural water use efficiency still had a negative effect on agricultural water use at a significance level of 1%, but the absolute value of the estimated coefficient increased relative to models 1 and 2. In Model 4, after rainfall was also added as the control variable, the agricultural water use efficiency still had a negative influence on agricultural water use at the significance level of 1%, and the absolute value of the estimated coefficient increased relative to Model 1 and 2, but decreased relative to Model 3. In Model 5, after all control variables (drought area, effective irrigation area, and rainfall) were added, the agricultural water use efficiency still had a negative effect on agricultural water use at the significance level of 1%, but the absolute value of the estimated coefficient decreased relative to Model 1, 3, and 4, but increased relative to Model 2. According to the estimated results of Model 5 including all the control variables, the agricultural water use can be reduced by 0.1119% if the agricultural water use efficiency increases by 1%, which means the agricultural water rebound effect is 88.81%. Thus, the agricultural water rebound effect with control variables is 3.33% higher than that without the control variables. In conclusion, regardless of the addition of control variables, agricultural water use efficiency has a significant negative impact on agricultural water use, but the water use cannot be reduced in equal proportion. These results verified Hypothesis 1 of this article.

Table 3 not only reflects the causal effect of agricultural water use efficiency on agricultural water use but also reflects the causal effect of control variables on agricultural water use. The control variables mainly include drought area, rainfall, and effective irrigation area. Drought area and rainfall are natural phenomena with strong exogeneity. The effective irrigated area is the result of the government’s construction of water conservancy facilities, which is exogenous, to a certain extent, and also related to drought area and rainfall. In other words, there is the problem of “self-selection” of policy implementation, but the causal relationship can be obtained by adding the control of drought area and rainfall. Both Model 2 and Model 5 show that the effective irrigated area has a positive influence on the agricultural water use at the significance level of 1%. According to the results of Model 5, the agricultural water use will increase by 0.4277% when the effective irrigated area increases by 1%. Both Model 3 and Model 5 show that the drought area has a positive impact on agricultural water use at a significance level of 1%. According to the results of Model 5, the agricultural water use will increase by 0.0067% when the drought area increases by 1%. Both Model 4 and Model 5 show that rainfall at a significance level of 1% had a negative impact on agricultural water use. According to Model 5, each 1% increase in rainfall will reduce agricultural water use by 0.0481%. In conclusion, the change in agricultural water use is not only related to agricultural water use efficiency but also affected by effective irrigation area, drought area, and rainfall. Hence, the additional agricultural water use could not just be attributed to the agricultural water use efficiency. However, the effective irrigation area, drought area, and rainfall should also be considered for the water saving effect after the government’s water-saving policy.

### 4.2. Heterogeneity

The estimated results in Table 3 show that agricultural water use efficiency has a significant negative impact on agricultural water use, leading to an average agricultural water rebound effect of 88.81%. The overall effect of agricultural water use efficiency on agricultural water use might mask heterogeneity of the impact. We explore the potential heterogeneity from two perspectives: rainfall size and whether it is a major grain-producing area.

The rainfall size reflects the average drought situation, and whether it is a major grain-producing area reflects the agricultural planting characteristics of the region. According to the amount of rainfall, the samples can be divided into two groups. One group is the samples with rainfall of 800 mm or less, which includes the arid, semi-arid, and sub-humid areas; the other group is the samples with rainfall of 800 mm or more, which includes the humid areas. The samples can be divided into two groups from the perspective of whether they are major grain-producing areas. The first group is the major grain-producing areas, including Liaoning, Hebei, Shandong, Jilin, Inner Mongolia, Jiangxi, Hunan, Sichuan, Hubei, Jiangsu, Henan, Anhui, and Heilongjiang. The other group is the non-grain main producing areas, including Beijing, Chongqing, Guangdong, Guangxi, Gansu, Hainan, Guizhou, Ningxia, Qinghai, Tianjin, Shanghai, Shaanxi, Shanxi, Xinjiang, Zhejiang, Yunnan, and Fujian. The above two groups reflect the drought situation and planting situation, respectively. The estimated results based on the heterogeneity analysis of the above groups are shown in Table 4.

The estimated results in Table 4 reflect the heterogeneity of the impact of agricultural water use efficiency on agricultural water use. Model 6 and Model 7 are the subsample estimation results of non-humid zone and humid zone, respectively. According to Model 6, agricultural water use efficiency has a negative impact on agricultural water use at a significance level of 1%. Specifically, when agricultural water use efficiency increases by 1%, agricultural water use will decrease by 0.0806%, which means the agricultural water rebound effect is 91.94%. According to Model 7, agricultural water use efficiency has a negative impact on agricultural water use at a significance level of 1%. Specifically, when agricultural water use efficiency increases by 1%, agricultural water use will decrease by 0.1463%, which means the agricultural water rebound effect is 85.37%. Therefore, the water-saving effect of the improvement of agricultural water use efficiency is worse under the relatively arid conditions than that under the relatively humid conditions. Thus, the agricultural water rebound effect will be greater in relatively arid regions.

Model 8 and Model 9 are the regression results for the subsample of major grain-producing areas and non-major grain-producing areas, respectively. According to Model 8, agricultural water use efficiency has a negative impact on agricultural water use at a significance level of 1%. Specifically, each 1% increase in agricultural water use efficiency will reduce agricultural water use by 0.1646%; that is, the agricultural water rebound effect is 83.54%. According to Model 7, agricultural water use efficiency has a negative impact on agricultural water use at a significance level of 5%. Specifically, when agricultural water use efficiency increases by 1%, agricultural water use will decrease by 0.0716%; that is, the agricultural water rebound effect is 92.84%. Therefore, compared with the major grain-producing areas, the agricultural water use efficiency in non-major grain-producing areas has a worse water-saving effect. Thus, the agricultural water rebound effect is also smaller in grain-producing areas.

In conclusion, in relatively arid regions, the improvement of agricultural water use efficiency has a greater agricultural water rebound effect. However, the improvement of agricultural water use efficiency in non-major grain-producing areas has worse water-saving effect than that in major grain-producing areas, leading to a greater agricultural water rebound effect in non-major grain-producing areas.

### 4.3. Mechanism

The estimation results in Table 3 show that agricultural water use efficiency has a significant negative impact on agricultural water use, but the mechanism needs to be further analyzed and verified. In order to verify that agricultural water use efficiency affects agricultural water use through planting structure and planting area, a mediating effect model was adopted to analyze it. Based on the method of the mediation model, further estimation is needed after the regression of Model 5. The mechanism results can be seen in Table 5. It should be noted that all the models in Table 5 adopt a fixed effect model and add all control variables, including drought area, effective irrigation area, and rainfall.

Model 10 and Model 11 in Table 5 show the influence of agricultural water use efficiency on the mediating variables (planting structure and planting area). The results of Model 10 show that the agricultural water use efficiency has a negative impact on the planting structure at a significance level of 5%. Specifically, when the agricultural water use efficiency increases by 1%, the proportion of grain planting will decrease by 0.0519%, which reflects that when the agricultural water use efficiency improves, the proportion of cash crops planted in each province will increase. The results of Model 11 show that the agricultural water use efficiency has a positive influence on the planting area at the significance level of 1%. Specifically, the planting area will increase by 0.0353%, if the agricultural water use efficiency increases by 1%, which reflects that the overall planting area of crops in each province will expand under the condition of the improvement of agricultural water use efficiency. In conclusion, agricultural water use efficiency will affect the crop planting structure and scale in the region, which may strengthen the agricultural production pattern in the region, and even lead to the formation of an agricultural production agglomeration.

According to the estimated results in Table 5, the mediating effect of agricultural water use efficiency on agricultural water use can be further reflected through planting structure and planting area. On the one hand, Model 10 and Model 11 reflect that agricultural water use efficiency can significantly affect the intermediate variables (planting structure and planting area). On the other hand, Model 12 and Model 13 reflect that the mediating variables (planting structure and planting area) also significantly affect agricultural water use. Specifically, Model 12 shows that when the planting structure increases by 1%, the agricultural water use will increase by 0.2220%. Model 13 shows that when the planting area increases by 1%, the agricultural water use will increase by 0.3346%. The results of the complete Model 14 are also basically consistent with the conclusions of Model 12 and Model 13. Therefore, it can be found that agricultural water use efficiency will affect agricultural water use through planting structure and planting area. These results verified our Hypothesis 2 of this article.

Model 14 in Table 5 also reflects the direct effect of agricultural water use efficiency on agricultural water use. Specifically, when agricultural water use efficiency increases by 1%, agricultural water use will directly decrease by 0.1128%. This is slightly higher than the net effect of agricultural water use efficiency on agricultural water use obtained from Model 5 (1% increase in agricultural water use efficiency will result in a net decrease of 0.1119% of agricultural water use). However, the difference between the direct effect and net effect is small. This is mainly reflected in the fact that the effects of agricultural water use efficiency on agricultural water use through planting structure and sown area are in opposite directions. Combined with Model 10 and Model 14, Model 10 shows that agricultural water use efficiency has a negative impact on planting structure, while Model 14 shows that planting structure has a positive impact on agricultural water use. Therefore, agricultural water use efficiency has a negative impact on agricultural water use through planting structure, so it can play a water-saving effect. Combined with Model 11 and Model 14, Model 11 shows that agricultural water use efficiency has a positive impact on sown area, while Model 14 shows that the sown area has a positive impact on agricultural water use. Therefore, agricultural water use efficiency has a positive impact on agricultural water use through the sown area, so it does not have the effect of saving water but increases the agricultural water use. Because of just these two different mechanisms, the net effect and direct effect of agricultural water use efficiency on agricultural water use are less different. The implication for agricultural water management is that the irrigation agricultural scale has to be controlled under the condition of available water resource while improving agricultural water use efficiency.

According to the results, it is reasonable to transfer grain crops to cash crops in the planting structure. However, the agricultural irrigation should be constrained on account of the water resource conditions because more planting areas will increase agricultural water demand for food production.

## 5. Discussion

### 5.1. Magnitude of the Rebound Effect

Most of the existing studies have focused on estimation of the agricultural rebound effect. The measures of the rebound effect are based on an assumption which is that a 1% increase in agricultural water efficiency requires a 1% reduction in agricultural water use. An agricultural water rebound effect between 0% and 100% can be called partial rebound, showing that partial potential agricultural water saving is offset in response to improvement of agricultural water use efficiency. If an agricultural water rebound effect is over 100%, the agricultural water use efficiency can increase the agricultural water use, which is also called the backfire effect. The main issue concerning the agricultural water rebound effect is that a backfire effect may exist if the agricultural water use efficiency can increase agricultural water use. Gómez and Pérez-Blanco [6] studied the conditions of Jevons paradox in water use through basic economic principles, and Pfeiffer and Lin [14] clearly state that a rebound effect of over 100% can occur.

Based on the results, the agricultural water use will decrease by 0.1119% if the agricultural water use efficiency increases by 1%. This means the average agricultural water rebound effect is 88.81% in China, which is greater than that in the results of Song et al. [7], Fang et al. [17], and Fei et al. [4]. Song et al. [7], Fang et al. [17] and Fei et al. [4] all focused on the magnitude of rebound effect in the agricultural sector with different methods. Song et al. [7] found the agricultural water rebound effect in China from 1998 to 2014 is 61.49%. Fang et al. [17] found that the average agricultural water rebound effect is 70.3% using data of China from 1998 to 2016. Fei et al. [4] found that the agricultural water use efficiency will offset 49.32% of the potential water saving in the short run and counteract 66.01% in the long run. Therefore, the agricultural water rebound effect may be more serious in China.

In this paper, it was also found that the relatively arid region experiences a greater agricultural water rebound effect than the relatively humid area in China, which corresponds with the results of Song et al. [7], but is different from the results of Fang et al. [17] on heterogeneity of the magnitude of rebound effect. The northern and western regions will be more arid than southern and eastern regions in China, and Song et al. [7] found that the northern and western regions of China experience a greater agricultural water rebound effect than the southern and eastern regions. However, Fang et al. [17] found that the agricultural water rebound effect in the southwest is the highest, whereas agricultural water rebound effect in the northwest is the lowest. Hence, heterogeneity of the agricultural water rebound effect does indeed exist in China.

In conclusion, the agricultural water rebound effect in this paper is greater than that in the results of studies on the magnitude of the rebound effect in China. Moreover, heterogeneity in the agricultural water rebound effect does indeed exist in China, and the agricultural water rebound effect will be greater in relatively arid areas. However, the agricultural water rebound effect is still less than 100%, indicating that the increase in agricultural water efficiency indeed results in water conservation to some extent. Hence, the backfire effect is not a serious issue in China’s agricultural sector.

### 5.2. Mechanism of the Rebound Effect

Existing studies have ignored the fact that the increase in agricultural water use may be caused by other factors. According to the results, the effective irrigated area, drought area, and rainfall can also lead to an increase in agricultural water use. The agricultural water use efficiency can affect agricultural water use through the planting structure and planting area. Moreover, the planting structure and planting area have opposite effects on the mechanism, in which the agricultural water use efficiency can increase the agricultural water use through planting area, but decrease the agricultural water use through planting structure.

Pfeiffer and Lin [14] found that the shift to more efficient irrigation technology has increased groundwater extraction in part due to shifting crop rotation patterns, which corresponds with the planting area in the mechanism because the crop rotation pattern is another way to increase the planting area. Loch and Adamson [5] found that greater technological change in irrigation will increase the higher use of land, then increase the water demand in Northern Murray–Darling Basin. Hence, this paper verifies the conclusion of Pfeiffer and Lin [14] and Loch and Adamson [5] that agricultural water use efficiency can increase agricultural water use through planting area.

Playán and Mateos [30] indicated that agricultural water use are bound because of the intensified cropping pattern with more water intensive crops (searching for economic efficiency), and other studies have reached the same conclusion that water rebound came from the introduction of new crops which are more water intensive after agricultural water use efficiency was improved [31]. However, in this paper, the increase in cash crops can reduce the amount of water used in agriculture, which is in contrast to Playán and Mateos [30] and Berbel et al. [31].

In conclusion, the mechanism through planting area is consistent with the existing research, but the mechanism through planting structure differs from the literature. Although the agricultural water use efficiency can affect the agricultural water use through planting area and planting structure, restraining the rebound effect of agricultural water focus on the planting area in China. The implication for agricultural water management in other regions is that the planting scale has to be controlled under the conditions of water resource.

## 6. Conclusions

Based on the panel data of 30 provinces in China from 2000 to 2017, the casual effect of agricultural water use efficiency on agricultural water use and the rebound effect of agricultural water use were empirically analyzed using a fixed model. Furthermore, the heterogeneity and mechanism of agricultural water use efficiency on agricultural water use was further analyzed.

The following conclusions can be drawn. First, agricultural water use efficiency has a significant negative impact on agricultural water use. Specifically, the agricultural water use can be reduced by 0.1119% for 1% increase in agricultural water use efficiency. This shows that the improvement in agricultural water use efficiency has a certain water-saving effect, but from the perspective of the rebound effect, there is an average of 88.81% rebound effect of agricultural water use. Second, the influence of agricultural water use efficiency on agricultural water use is heterogeneous. Compared with humid areas, the water saving effect of the improvement in agricultural water use efficiency is worse in relatively arid areas, and the rebound effect of agricultural water use is greater. Compared with the major grain-producing areas, the water-saving effect of the improvement in agricultural water use efficiency is relatively worse in non-major grain-producing areas, and the rebound effect of agricultural water use is also greater in non-major grain-producing areas. Third, agricultural water use efficiency can affect agricultural water use through planting structure and planting area. The agricultural water use efficiency will reduce the agricultural water use by reducing the proportion of grain planted, while the agricultural water use efficiency will increase the agricultural water use by increasing the planting area. As a result, the net and direct effects of the increase in agricultural water use efficiency on agricultural water use are almost the same.

Although the rebound effect of agricultural water use indeed exists, the improvement of agricultural water use efficiency can indeed bring about the reduction of agricultural water use, but it cannot be realized that a 1% increase in agricultural water use efficiency can bring about a 1% decrease in agricultural water use. Therefore, the agricultural water-saving policy carried out by the government is still an important way to alleviate the water scarcity. However, while promoting agricultural water-saving policies to improve agricultural water use efficiency, other supporting policies are also needed. To be specific, on the one hand, it is reasonable to transfer grain crops to cash crops in the planting structure and constrain planting areas on account of the water resource conditions. On the other hand, the total water use in arid and non-major grain-producing areas should be controlled while improving agricultural water use efficiency.

## Figures and Tables

**Table 1:** The detailed grouping results of evaluated provinces.

Eastern	Central	Western
Beijing	Anhui	Chongqing
Guangdong	Guangxi	Gansu
Hainan	Heilongjiang	Guizhou
Hebei	Hubei	Ningxia
Liaoning	Hunan	Qinghai
Tianjin	Jilin	Sichuan
Shanghai	Inner Mongolia	Shaanxi
Shandong	Shanxi	Xinjiang
Zhejiang	Jiangxi	Yunnan
Jiangsu	Henan	
Fujian		

**Table 2:** Descriptive statistics of the selected variables.

Variables	Mean	Std. Dev.	Min	Max
WUE (Percent, %)	46.95	27.01	3.90	100.00
UE (Hundred million cubic meters)	119.99	100.37	3.3	561.75
EIA (Thousands hectares)	1964.42	1519.42	103.92	6208.23
RF (Millimeters)	891.41	527.75	36.6	2678.9
DA (Thousands hectares)	584	813.4	0	6500
PS (Percent, %)	61.05	18.49	7.04	97.64
PA (Thousands hectares)	5163.06	3642.89	88.6	14,783.4

**Table 3:** Estimation results.

	Model 1	Model 2	Model 3	Model 4	Model 5
Variables	lnWU	lnWU	lnWU	lnWU	lnWU
lnWUE	−0.1452 ***	−0.0977 ***	−0.1576 ***	−0.1497 ***	−0.1119 ***
	(−6.1948)	(−4.7173)	(−6.6302)	(−6.4394)	(−5.4094)
lnEIA		0.4243 ***			0.4277 ***
		(12.8558)			(13.1999)
lnDA			0.0076 ***		0.0067 ***
			(2.6457)		(2.6148)
lnRF				−0.0595 ***	−0.0481 ***
				(−3.3286)	(−2.9983)
Constant	4.9734 ***	1.7329 ***	4.9798 ***	5.3832 ***	2.0434 ***
	(58.2394)	(6.5949)	(58.6333)	(36.0412)	(7.2646)
N	540	540	540	540	540
Within $R^{2}$	0.0701	0.2984	0.0827	0.09	0.3274

Note: The above models are all fixed effect models; the values in parentheses are *t* values; *** means significant at the 1% level.

**Table 4:** Results of heterogeneity.

	Model 6	Model 7	Model 8	Model 9
Variables	lnWU	lnWU	lnWU	lnWU
lnWUE	−0.0806 ***	−0.1463 ***	−0.1646 ***	−0.0716 **
	(−2.7337)	(−5.3534)	(−5.7399)	(−2.3737)
lnEIA	0.5395 ***	0.1183 *	0.3855 ***	0.4164 ***
	(14.1665)	(1.8709)	(7.8503)	(9.3592)
lnDA	0.0057	0.0012	0.0018	0.0113 ***
	(1.3858)	(0.3467)	(0.553)	(2.7523)
lnRF	−0.0362 *	−0.1078 ***	−0.0417 **	−0.0476 *
	(−1.8143)	(−3.2989)	(−2.1653)	(−1.7734)
Constant	0.8861 ***	5.0432 ***	2.7924 ***	1.7785 ***
	(2.621)	(9.5641)	(6.0629)	(4.8466)
N	277	263	234	306
Subsample	Non−Humid	Humid	Grain	Non−Grain
Within $R^{2}$	0.4907	0.1592	0.4152	0.2871

Note: The above models are all fixed effect models; the values in parentheses are *t* values; *** means significant at the 1% level, ** means significant at the 5% level, and * means significant at the 1% level; “Non-Humid” means non-humid areas, which refers to the areas with annual average rainfall of less than 800 mm, while “Humid” means the humid area, which refers to the area with annual average rainfall of over 800 mm; “Grain” means major grain-producing areas including Liaoning, Hebei, Shandong, Jilin, Inner Mongolia, Jiangxi, Hunan, Sichuan, Hubei, Jiangsu, Henan, Anhui, and Heilongjiang, while “Non-Grain” means the non-major grain-producing areas including Beijing, Chongqing, Guangdong, Guangxi, Gansu, Hainan, Guizhou, Ningxia, Qinghai, Tianjin, Shanghai, Shaanxi, Shanxi, Xinjiang, Zhejiang, Yunnan, and Fujian.

**Table 5:** The mechanism results.

	Model 10	Model 11	Model 12	Model 13	Model 14
Variables	lnPS	lnPA	lnWU	lnWU	lnWU
lnWUE	−0.0519 **	0.0353 **	−0.1004 ***	−0.1237 ***	−0.1128 ***
	(−2.5779)	(2.0221)	(−4.9327)	(−6.2023)	(−5.7191)
lnEIA	0.0004	0.4898 ***	0.4276 ***	0.2638 ***	0.2744 ***
	(0.0142)	(17.9194)	(13.5025)	(6.6321)	(7.0175)
lnDA	0.0043 *	0.003	0.0058 **	0.0057 **	0.0049 **
	(1.7061)	(1.3946)	(2.2918)	(2.3087)	(2.0306)
lnRF	−0.0117	−0.0299 **	−0.0455 ***	−0.0381 **	−0.0364 **
	(−0.7508)	(−2.2101)	(−2.9002)	(−2.4609)	(−2.3990)
lnPS			0.2220 ***		0.1959 ***
			(4.9713)		(4.5278)
lnPA				0.3346 ***	0.3128 ***
				(6.6109)	(6.2693)
Constant	4.2525 ***	4.7097 ***	1.0991 ***	0.4677	−0.2627
	(15.5418)	(19.8489)	(3.2893)	(1.2983)	(−0.6762)
N	540	540	540	540	540
Within $R^{2}$	0.0188	0.3943	0.3588	0.381	0.4052

Note: The above models are all fixed effect models; the values in parentheses are *t* values; *** means significant at the 1% level, ** means significant at the 5% level, and * means significant at the 1% level.

## Data Availability

Data are available on request due to restrictions, e.g., privacy or ethical. The data presented in this study are available on request from the corresponding author. The data are not publicly available due to the strict management of various data and technical resources within the research teams.

## References

[1] Wheeler S.A., Carmody E., Grafton R.Q., Kingsford R.T., Zuo A. (2020). The rebound effect on water extraction from subsidising irrigation infrastructure in Australia. Resour. Conserv. Recycl..

[2] Yao L., Zhao M., Xu T. (2017). China’s water-saving irrigation management system: Policy, implementation, and challenge. Sustainability.

[3] Grafton R.Q., Williams J., Perry C.J., Molle F., Ringler C., Steduto P., Udall B., Wheeler S.A., Wang Y., Garrick D. (2018). The paradox of irrigation efficiency. Science.

[4] Fei R., Xie M., Wei X., Ma D. (2021). Has the water rights system reform restrained the water rebound effect? Empirical analysis from China’s agricultural sector. Agric. Water Manag..

[5] Loch A., Adamson D. (2015). Drought and the rebound effect: A Murray–Darling Basin example. Nat. Hazards.

[6] Gómez C.M., Pérez-Blanco C.D. (2014). Simple myths and basic maths about greening irrigation. Water Resour. Manag..

[7] Song J., Guo Y., Wu P., Sun S. (2018). The agricultural water rebound effect in China. Ecol. Econ..

[8] Ward F.A., Pulido-Velazquez M. (2008). Water conservation in irrigation can increase water use. Proc. Natl. Acad. Sci. USA.

[9] Peterson J.M., Ding Y. (2005). Economic adjustments to groundwater depletion in the high plains: Do water-saving irrigation systems save water?. Am. J. Agric. Econ..

[10] Fishman R., Devineni N., Raman S. (2015). Can improved agricultural water use efficiency save India’s groundwater?. Environ. Res. Lett..

[11] Yang Q., Zhu Y., Wang F. (2021). Exploring Mediating Factors between Agricultural Training and Farmers’ Adoption of Drip Fertigation System: Evidence from Banana Farmers in China. Water.

[12] Koundouri P., Nauges C., Tzouvelekas V. (2006). Technology adoption under production uncertainty: Theory and application to irrigation technology. Am. J. Agric. Econ..

[13] Dridi C., Khanna M. (2005). Irrigation technology adoption and gains from water trading under asymmetric information. Am. J. Agric. Econ..

[14] Pfeiffer L., Lin C.Y.C. (2014). Does efficient irrigation technology lead to reduced groundwater extraction? Empirical evidence. J. Environ. Econ. Manag..

[15] Li H., Zhao J. (2018). Rebound effects of new irrigation technologies: The role of water rights. Am. J. Agric. Econ..

[16] Berbel J., Mateos L. (2014). Does investment in irrigation technology necessarily generate rebound effects? A simulation analysis based on an agro-economic model. Agric. Syst..

[17] Fang L., Wu F., Yu Y., Zhang L. (2020). Irrigation technology and water rebound in China’s agricultural sector. J. Ind. Ecol..

[18] Ajaz A., Datta S., Stoodley S. (2020). High Plains Aquifer–State of Affairs of Irrigated Agriculture and Role of Irrigation in the Sustainability Paradigm. Sustainability.

[19] Brinegar H.R., Ward F.A. (2009). Basin impacts of irrigation water conservation policy. Ecol. Econ..

[20] Dagnino M., Ward F.A. (2012). Economics of agricultural water conservation: Empirical analysis and policy implications. Int. J. Water Resour. Dev..

[21] Kuil L., Evans T., McCord P.F., Salinas J.L., Bloeschl G. (2018). Exploring the influence of smallholders’ perceptions regarding water availability on crop choice and water allocation through socio-hydrological modeling. Water Resour. Res..

[22] Lecina S., Isidoro D., Playan E., Aragues R. (2010). Irrigation modernization and water conservation in Spain: The case of Riegos del Alto Aragon. Agric. Water Manag..

[23] Lopez-Gunn E., Zorrilla P., Prieto F., Llamas M.R. (2012). Lost in translation? Water efficiency in Spanish agriculture. Agric. Water Manag..

[24] Perry C. (2007). Efficient irrigation; inefficient communication; flawed recommendations. Irrig. Drain..

[25] Qureshi M.E., Schwabe K., Connor J., Kirby M. (2010). Environmental water incentive policy and return flows. Water Resour. Res..

[26] Scheierling S.M., Young R.A., Cardon G.E. (2006). Public subsidies for water-conserving irrigation investments: Hydrologic, agronomic, and economic assessment. Water Resour. Res..

[27] Sears L., Caparelli J., Lee C., Pan D., Strandberg G., Vuu L., Lawell C.Y.C.L. (2018). Jevons’ paradox and efficient irrigation technology. Sustainability.

[28] Wu F., Zhang Q., Gao X. (2018). Does water-saving technology reduce water use in economic systems? A rebound effect in Zhangye city in the Heihe River Basin, China. Water Policy.

[29] Freire-González J. (2019). Does water efficiency reduce water consumption? The economy-wide water rebound effect. Water Resour. Manag..

[30] Playán E., Mateos L. (2006). Modernization and optimization of irrigation systems to increase water productivity. Agric. Water Manag..

[31] Berbel J., Gutiérrez-Martín C. (2015). Literature review on rebound effect of water saving measures and analysis of a Spanish case study. Water Resour. Manag..

[32] Berkhout P.H.G., Muskens J.C., Velthuijsen J.W. (2000). Defining the rebound effect. Energy Policy.

[33] Freire-González J. (2011). Methods to empirically estimate direct and indirect rebound effect of energy-saving technological changes in households. Ecol. Model..

[34] Adetutu M.O., Glass A.J., Weyman-Jones T.G. (2016). Economy-wide estimates of rebound effects: Evidence from panel data. Energ. J..

[35] Ouyang J., Long E., Hokao K. (2010). Rebound effect in Chinese household energy efficiency and solution for mitigating it. Energy.

[36] Saunders H.D. (2010). A view from the macro side: Rebound, backfire, and Khazzoom–Brookes. Energy Policy.

[37] Sorrell S., Dimitropoulos J. (2010). The rebound effect: Microeconomic definitions, limitations and extensions. Ecol. Econ..

[38] Wang Z., Lu M. (2014). An empirical study of direct rebound effect for road freight transport in China. Appl. Energy.

[39] Perry C. (2011). Accounting for water use: Terminology and implications for saving water and increasing production. Agric. Water Manag..

[40] Contor B.A., Taylor R.G. (2013). Why improving irrigation efficiency increases total volume of consumptive use. Irrig. Drain..

[41] Huffaker R., Whittlesey N. (2000). The allocative efficiency and conservation potential of water laws encouraging investments in on-farm irrigation technology. Agric. Econ..

[42] García-Garizábal I., Causapé J. (2010). Influence of irrigation water management on the quantity and quality of irrigation return flows. J. Hydrol..

[43] Camacho Poyato E., Montesinos Barrios M.P., Pérez Urrestarazu L., Rodríguez Díaz J.A. (2011). The paradox of irrigation scheme modernization: More efficient water use linked to higher energy demand. Span. J. Agric. Res..

[44] Bai C., Feng C., Du K., Wang Y., Gong Y. (2020). Understanding spatial-temporal evolution of renewable energy technology innovation in China: Evidence from convergence analysis. Energy Policy.

[45] Feng C., Wang M. (2017). Analysis of energy efficiency and energy savings potential in China’s provincial industrial sectors. J. Clean. Prod..

[46] Li J., Liu H., Du K. (2019). Does market-oriented reform increase energy rebound effect? Evidence from China’s regional development. China Econ. Rev..

[47] Tian S., Xu L., Wu X. (2021). Impacts of Social Participation on Self-Rated Health of Aging Women in China: With a Mediating Role of Caring for Grandchildren. Int. J. Environ. Res. Public Health.

[48] Usán Supervía P., Quílez Robres A. (2021). Emotional Regulation and Academic Performance in the Academic Context: The Mediating Role of Self-Efficacy in Secondary Education Students. Int. J. Environ. Res. Public Health.

